# Influence of the Quaternary Glacial Cycles and the Mountains on the Reticulations in the Subsection *Willkommia* of the Genus *Centaurea*

**DOI:** 10.3389/fpls.2019.00303

**Published:** 2019-03-21

**Authors:** Samira Ben-Menni Schuler, Jordi López-Pujol, Gabriel Blanca, Roser Vilatersana, Núria Garcia-Jacas, Víctor N. Suárez-Santiago

**Affiliations:** ^1^Departamento de Botánica, Facultad de Ciencias, Universidad de Granada, Granada, Spain; ^2^Botanic Institute of Barcelona (IBB, CSIC-ICUB), Barcelona, Spain

**Keywords:** *Centaurea*, gene flow, Quaternary glaciations, reticulate evolution, secondary contacts

## Abstract

Late Neogene and Quaternary climatic oscillations have greatly shaped the genetic structure of the Mediterranean Basin flora, with mountain plant species tracking warm interglacials/cold glacials by means of altitudinal shifts instead of broad latitudinal ones. Such dynamics may have enhanced population divergence but also secondary contacts. In this paper, we use a case example of subsection *Willkommia* of *Centaurea* (comprising three narrowly distributed endemic species, *Centaurea gadorensis*, *C. pulvinata*, and *C. sagredoi*) to test for reticulate evolution and recurrent hybridizations between nearby populations. For this, we combine analyses of genetic diversity and structuring, gene flow and spatial correlation, and ecological niche modeling. Our results support the contention that the current genetic structure of the three species is the result of historical gene flow at sites of secondary contact during the glacial periods, followed by isolation after the retraction of populations to the middle-upper areas of the mountains during the interglacial periods. The extent and direction of the gene flow was determined largely by the location of the populations on mountainsides oriented toward the same valley or toward different valleys, suggesting the intermountain valleys as the areas where secondary contacts occurred.

## Introduction

Climatic changes since the end of the Neogene have greatly modeled the flora of the Mediterranean Basin in terms of species distributions and divergence (Bocquet et al., 1978; Blondel and Aronson, 1995; Hewitt, 1996; Gómez and Lunt, 2007). Pleistocene glaciations caused some of the disjunct distribution patterns found in several groups of Mediterranean plants and have shaped their population genetic structure (Hewitt, 1996, 1999, 2000; Taberlet et al., 1998; Petit et al., 2003, 2005; Svenning and Skov, 2007). Specifically, one of the most common plant-distribution patterns in the Mediterranean mountain ranges is schizo-endemism (e.g., Verlaque et al., 1997; Thompson, 2005), in which fragmentation and isolation of a widespread ancestral taxon favored allopatric differentiation of range-restricted endemic species. The resulting species are closely related, share the same chromosome number, but inhabit disjunct areas (Favarger and Contandriopoulos, 1961).

During the Pleistocene, mountains in the Mediterranean peninsulas acted as glacial refugia (Taberlet et al., 1998; Hewitt, 1999; Petit et al., 2003; Médail and Diadema, 2009). Mediterranean refugia are usually regarded as “southern refugia” (*sensu* Stewart et al., 2010) or “macrorefugia” (*sensu* Rull, 2009), as they constituted, in most cases, the source for further northward recolonization. Such migration events usually implied large latitudinal movements (of hundreds of kilometers) and occurred through multiple founding events, resulting in the pattern widely known as “southern richness vs. northern purity” (Hewitt, 2000). This basic model, however, may be more complex because of the persistence of species throughout the glacial periods in refugia at latitudes higher than those of the southern refugia, from which they recolonized surrounding areas as the climate improved (Stewart and Lister, 2001). Plant populations that remained in the southern European mountains, in contrast, survived warm interglacials/cold glacials by means of altitudinal shifts (Hewitt, 2000). It is generally accepted that the wide diversity of microhabitats throughout the rugged topography of these areas allowed species to migrate along altitudinal gradients, favoring persistence, but also differentiation of isolated populations in different mountains or even at short distances in the same mountain range (Hewitt, 1999; Gómez and Lunt, 2007; Médail and Diadema, 2009; Jiménez-Mejías et al., 2015). However, vertical movements induced by the repeated glacial/interglacial cycles of the Pleistocene could also have encouraged hybridization and reticulate evolution in lineages from Mediterranean mountains. At lower altitudes, secondary contacts between vicariant lineages occurred over the course of different glacial maxima, facilitating gene flow, hybridization, and hybrid speciation, resulting in complex evolutionary patterns (Thompson, 2005; Nieto Feliner, 2011, 2014). The reticulate evolution pattern linked to altitudinal shifts has been documented in several Mediterranean genera (e.g., Fuertes Aguilar et al., 1999; Albaladejo and Aparicio, 2007; Suárez-Santiago et al., 2007a; Alarcón et al., 2012; Maguilla and Escudero, 2016) including *Centaurea* (Suárez-Santiago et al., 2007b; Garcia-Jacas et al., 2009; López-Vinyallonga et al., 2015; López-Pujol et al., 2016).

One of the groups in which reticulation phenomena have been detected is subsection *Willkommia* (Blanca) Garcia-Jacas, Hilpold, Susanna, and Vilatersana of the genus *Centaurea* L. (Asteraceae; Suárez-Santiago et al., 2007b). This subsection includes 20 species and 18 subspecies endemic to the eastern Iberian Peninsula and northwestern Africa (Breitwieser and Podlech, 1986; Devesa et al., 2014; Hilpold et al., 2014a). The origin of *Willkommia* has been dated to about 5.5 Ma (Suárez-Santiago et al., 2007b). Until recently, diversification within this subsection was attributed to schizo-endemism processes (Blanca, 1981a). Nevertheless, the additivity patterns of nuclear ribosomal ITS and ETS sequences and the geographical structure of the detected ribotypes (Suárez-Santiago, 2005; Suárez-Santiago et al., 2007b), as well as the evolutionary analysis of a satellite-DNA family within the subsection (Suárez-Santiago et al., 2007c) clearly indicate that *Willkommia* diversification has followed a model of reticulate evolution. This reticulation is thought to be the consequence of recurrent hybridizations between divergent populations within the geographical range of a primary radiation (i.e., microallopatric), triggered by the Pleistocene climatic oscillations in the complex local topography (Suárez-Santiago et al., 2007b).

Reticulate evolution is usually detected through phylogenetic analyses based on nucleotide sequences of multiple species, as was the case in the subsection *Willkommia*. However, to unravel the reticulation process and its consequences on the genetic structure and the identity of the species, more fine-scale studies are needed (Nieto Feliner, 2011). Phylogeographic analysis of population differentiation within species and among closely related species within a geographically restricted area enables the testing of a specific hypothesis on the evolutionary pattern followed by a group of plant species and also makes it possible to infer the role of climate changes as well as topography in explaining such evolutionary patterns.

**FIGURE 1:**
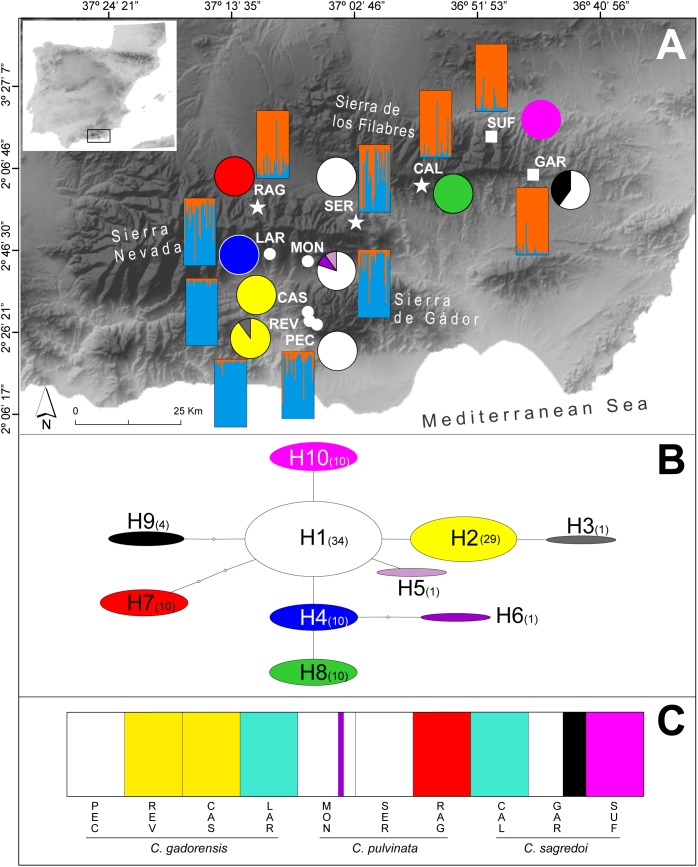
Distribution and genetic composition of the populations sampled. **(A)** Geographical distribution of the 10 populations of *C. gadorensis* (circles), *C. pulvinata* (stars), and *C. sagredoi* (squares), distribution of the cpDNA haplotypes (pie charts), and estimated genetic structure based on microsatellite data using the Bayesian approach implemented in STRUCTURE (bar plots). Pie charts indicate haplotype frequencies. STRUCTURE analysis is shown for *K* = 2, which represents the uppermost hierarchical level estimated. **(B)** Inferred cpDNA network, following the statistical parsimony method, with TCS. Circle sizes are proportional to the haplotype frequencies. Numbers in parenthesis indicate how many individuals have each haplotype. **(C)** Bar plot showing the results of the Bayesian clustering of cpDNA data implemented in BAPS. Population codes and species names are shown below. For population codes, see Supplementary Table 1.

In this study, we have selected a case example within the subsection *Willkommia* comprising three narrowly distributed endemic species: *Centaurea gadorensis* Blanca, *C. pulvinata* (Blanca) Blanca, and *C. sagredoi* Blanca. Phylogenetic analyses have shown that *C. pulvinata* and *C. sagredoi* are sister species, while *C. gadorensis* is genetically differentiated from them (Suárez-Santiago et al., 2007b). However, their relationships have not been completely resolved, mainly due to the polymorphisms found in the ITS/ETS sequences of *C. pulvinata*, which show an additivity pattern with respect to the other two species, suggesting gene flow during diversification (Suárez-Santiago, 2005). Morphologically, the three species are closely related and are differentiated mainly by characters related to the habit of the plant, upper cauline leaves, involucre, and pappus of the achene (see Supplementary Figure 1 and, for more details on the taxonomy and distribution of these species, the review in *Flora iberica* by Devesa et al., 2014). All three species are distributed within a mountain area that comprises three close massifs in the Baetic System of southern Spain (Devesa et al., 2014) (Figure 1A): Sierra de los Filabres, Sierra Nevada, and Sierra de Gádor. Also, all three are located totally or partially within the Sierra Nevada/Gata area, one of the main Mediterranean plant refugia (Médail and Diadema, 2009). *Centaurea sagredoi* is endemic to the northernmost of the three massifs, the Sierra de los Filabres, and it grows on acidic soils (schists). Only two populations are known, one on the northern mountainside and the other on the southern mountainside. *Centaurea gadorensis* can be found in the southernmost mountain range, the Sierra de Gádor, where up to seven populations have been reported on basic soils (limestone). However, two populations of this species are located on the southern side of Sierra Nevada, a large massif situated geographically between Sierra de los Filabres and Sierra de Gádor on schists (Figure 1A). Finally, *Centaurea pulvinata* is restricted to two large populations along the northern mountainsides of Sierra Nevada and one population on the southern mountainside of Sierra de los Filabres, in all cases on acidic substrates (Figure 1A).

The geographically restricted three-species system allows us to investigate the reticulate evolution proposed in the subsection *Willkommia* and how this was favored by the Pleistocene climate fluctuations and topography. Here, we test whether the three species originated in a context of vicariance interrupted by secondary contacts and hybridization among the three species, or in a scenario in which hybridization played no role during speciation. Regarding reticulation in the evolution of the three species, we evaluate the role of the topography and climatic fluctuations of the Pleistocene in favoring secondary contacts in lowlands between nearby populations oriented toward the same valley. To test these hypotheses, we undertake the following tasks: (1) we study the population genetic differentiation at intra- and interspecific level using different methodological approaches and thus gather evidence of the reticulate evolution pattern they could have followed; (2) we examine suitable distributions for the species at the present and the Last Glacial Maximum (LGM; *c*. 21 ka); 3) we determine how geographical, climatic, and topographic variation over space and time explain the pattern of population genetic structure.

For these tasks, we use two complementary approaches. First, for the genetic study, we analyze the genetic variation using a combination of two different molecular markers with different evolutionary rates and modes of inheritance: nuclear loci for microsatellites, and nucleotide sequences of the intergenic spacer *trn*T–*trn*L of the chloroplast DNA (cpDNA). Second, for the distribution study, we use ecological niche modeling (ENM) to build the specific paleodistribution models.

## Materials and Methods

### Plant Material

A total of 10 populations were sampled throughout the distribution range of the three species (Figure 1A and Supplementary Table 1). These included the only known populations of *C. sagredoi* and *C. pulvinata* (two and three populations, respectively), and five populations of *C. gadorensis* (three from Sierra de Gádor and two from Sierra Nevada). The populations were taxonomically assigned to each of the three species according to the review of the genus in the Iberian Peninsula (Devesa et al., 2014). All the populations except two of *C. gadorensis* (PEC and REV) were sampled in early summer of 2012. To increase the sampling of *C. gadorensis* in the Sierra de Gádor, we sampled the PEC and REV populations in the summer of 2014. For each population, leaf samples from 30 adult individuals (i.e., 300 individuals in total) were collected. The individuals were randomly collected, trying to cover the maximum possible area of each population while maintaining a minimum distance of one meter between individuals.

### DNA Extraction, Microsatellite Genotyping, and cpDNA Sequencing

For microsatellite analyses, the genomic DNA of the 300 sampled individuals (30 individuals of 10 populations) was extracted from silica-dried leaves using the kit NucleoSpin Plant II (Macherey-Nagel), following the manufacturer’s instructions. All individuals were genotyped for seven microsatellite loci as described in Fréville et al. (2000; loci: *13D10*, *21D9*) and in Marrs et al. (2006; loci: *CD37*, *42CM27*, *25CM6*, *CM17*, *CM15*), and following the PCR conditions explained in those studies. We selected these loci from all the ones described in Fréville et al. (2000) and Marrs et al. (2006) because they were the only ones that yielded an unambiguous amplification pattern. Genotyping was performed on an ABI PRISM^^®^^ 3100-Avant Genetic Analyzer (Applied Biosystems, Foster City, CA, United States). Alleles were scored using GENEMARKER v1.85 (SoftGenetics, State College, PA, United States).

The plastid intergenic spacer *trn*T–*trn*L was amplified and sequenced for a subsample of 10 individuals per population (100 individuals in total), using the primers *a* and *b* (Taberlet et al., 1991), and the PCR conditions in Small et al. (1998). Sequencing was performed on an ABI PRISM^^®^^ 3100-Avant Genetic Analyzer (Applied Biosystems, Foster City, CA, United States). The resulting sequences were aligned using the Clustal algorithm in the alignment editor BIOEDIT v7.0.5.3 (Hall, 1999), and then adjusted by eye. Indels were coded as presence or absence at the end of the data matrix following the simple indel coding method (Simmons and Ochoterena, 2000), as implemented in INDELCODER (Ogden and Rosenberg, 2007).

The genotype matrix used in this article is available on Figshare at https://figshare.com/s/4c7e64826f27b05499f2. All cpDNA sequences were deposited in European Nucleotide Archive (accessions LS974119–LS974128).

### Genetic Diversity and Structure

Genotypic linkage disequilibrium and the departure from Hardy-Weinberg expectations were tested with FSTAT v2.9.4 (Goudet, 2003) and GENODIVE v2.0b24 (Meirmans and Van Tienderen, 2004), respectively. The frequency of null alleles at each locus and for each population, and the inbreeding coefficients (*f*) were estimated with the software INEST v2.2 (Chybicki and Burczyk, 2009). INEST takes into account simultaneously null allele frequencies at each locus and the average level of the intrapopulation inbreeding as a multilocus parameter. We used the Bayesian method proposed by Vogl et al. (2002), which provides robust estimates for multi-locus microsatellite data even in the presence of null alleles (Chybicki and Burczyk, 2009). To test whether the excess of homozygotes was due to inbreeding, we compared the full model (used to estimate *f* considering null alleles) and a null model (*f* as constants equal 0) using the Deviance Information Criterion (DIC) (Spiegelhalter et al., 2002). The model with the lowest DIC will be the model best fit to the data. When the full model fits the data better, it means that inbreeding is the most important component of the model, and explains the high value of *f*. INEST was also used to calculate the observed and expected heterozygosities (*H*_O_, *H*_E_) corrected for null alleles. GENODIVE was used to estimate the mean number of alleles per locus (*A*) and the number of private alleles. Allelic richness (*Ar*) was calculated using FSTAT. All these statistics were computed for each population and for each species (by pooling populations) across all loci. When multiple tests were involved, the sequential Bonferroni-type correction was applied to test for significance (Rice, 1989). For cpDNA, nucleotide (π) and haplotype (*H*) diversity were calculated using ARLEQUIN v3.5.2.2 (Excoffier and Lischer, 2010).

The distribution of genetic variability within and among populations, and among species, was evaluated for all molecular markers using an analysis of molecular variance (AMOVA; Excoffier et al., 1992) and tested with a permutation test (10,000 permutations) with ARLEQUIN. Population genetic structure was analyzed using different approaches. First, *F*_ST_ values were calculated for each pair of species and populations using GENODIVE, for microsatellite data, and for each pair of species with ARLEQUIN for cpDNA. Second, the Bayesian algorithm implemented in STRUCTURE v2.3.3 (Falush et al., 2003) was used to evaluate the number of genetic clusters (*K*) in our microsatellite data. The number of clusters tested ranged from one to 11, with 10 replicates per *K*, using the admixture model and correlated allele frequencies. The burn-in period and Markov Chain Monte Carlo (MCMC) iterations were set to 50,000 and 10^6^, respectively. The optimal number of clusters was estimated with the online tool STRUCTURESELECTOR (Li and Liu, 2018). We identified the uppermost hierarchical level of genetic structure using the delta *K*-method (Δ*K*; Evanno et al., 2005), which accurately identifies it when the populations are evenly sampled (Puechmaille, 2016); as is the case here. To explore other levels of genetic partitioning, we used the mean posterior probabilities lnP(*K*) (Pritchard et al., 2000) and the four independent estimators proposed by Puechmaille (2016; MedMedK, MedMeaK, MaxMedK, and MaxMeaK) considering a membership coefficient threshold of 0.5. To align and visualize the STRUCTURE output across the 10 replicates, we used the online program CLUMPAK (Kopelman et al., 2015). Third, for microsatellites, the genetic structure was also assessed using a model-free multivariate statistics-based clustering method, a discriminant analysis of principal components (DAPC) on R package ADEGENET (Jombart et al., 2010). The function *xvalDapc* from ADEGENET was used to select by cross-validation the correct number of principal components with 1,000 replicates using a training set of 90% of the data. The number of principal components was chosen based on the criteria that it had to produce the highest average percentage of successful reassignment and lowest root mean squared error (Jombart et al., 2010). Finally, BARRIER v2.2 (Manni et al., 2004) was used to identify sharp genetic breaks among populations based on Monmonier’s algorithm; such genetic “barriers” are often interpreted as depicting the geographical location of putative landscape features that influence gene flow (Manni et al., 2004). The significance of our calculations was tested by means of 1,000 bootstrap matrices of Nei’s genetic distance *D*_a_ (Nei et al., 1983) previously established with MICROSATELLITE ANALYZER (MSA) v4.05 software (Dieringer and Schlötterer, 2003). In this way, up to 10 barriers were tested.

It was found that only locus *21D9* has a moderate null allele frequency (mean = 0.157), while the rest has a mean frequency < 0.05 (see the section “Results”). To test the possible effect of the null alleles at the *21D9* locus on *F*_ST_ values and genetic structuring analyses, we repeated some of these analyses, considering null alleles (pairwise *F*_ST_ using the software FreeNA; Chapuis and Estoup, 2007) and eliminating this locus (AMOVA, STRUCTURE). The results were almost equal when null alleles are not considered and when *21D9* is included (data not shown), so this locus was not expected to cause significant problems in the analyses. It has been suggested that biases are negligible when null alleles are present at frequencies below 0.200 (Dakin and Avise, 2004). Therefore, we kept *21D9* in all analyses.

A cpDNA network was reconstructed following the statistical parsimony method (Templeton et al., 1992) as implemented in TCS v1.21 (Clement et al., 2000). Moreover, a Bayesian clustering of cpDNA data was implemented in BAPS v6.0 (Corander et al., 2008) to analyze the population genetic structure by clustering sampled individuals into groups. We ran 10 replicates from each of the 11 simulations from *K* = 1 to *K* = 11 during mixture analyses. The parameters for admixture analyses based on the mixture analyses were set as recommended in the BAPS manual (100 iterations used to estimate the admixture coefficients for the individuals, 200 reference individuals, and 20 iterations used to estimate the admixture coefficients for the reference individuals; Corander et al., 2013).

### Gene Flow and Spatial Correlation

A number of independent tests were carried out using the microsatellite dataset to investigate how gene flow has shaped genetic diversity and population structure of the species studied. First, to test whether the divergence process between the species fits the schizo-endemic pattern proposed for subsection *Willkommia* or whether there was gene flow between populations and species during their divergence, we used the MCMC approach implemented in the program 2MOD v0.2 (Ciofi et al., 1999). The method compares the relative likelihoods for two contrasting models of demographic history, a model of immigration-drift equilibrium (gene flow model) vs. ancestral population fragmentation into independent units diverging by drift (drift model). The program was run with MCMC simulation of 1,000,000 iterations; 10% of the output was discarded as the burn-in period. Probabilities of each model were calculated using both the species and the sampling locality as population units. We used Bayes factors (BF) to describe the probability of the most likely model over the probability of the other model.

Second, we tested the connectivity among populations by estimating the migration rates among them. Thus, to know whether there was recent (over two to three generations) gene flow between the populations, we estimated migration rates (*m*) between all individual populations using a Bayesian assignment test with the software BAYESASS v1.3 (Wilson and Rannala, 2003). As program settings, the default values were used (MCMC iterations, 3 × 10^6^; length of the burn-in, 999,999; sampling frequency, 2,000; delta value, 0.15). In addition, the extent and direction of historical gene flow among populations were estimated by calculating the historical mutation-scale migration rates (*M*) with MIGRATE-N v3.6.4 (Beerli and Felsenstein, 2001). We ran 30 replicates under a Brownian motion model, assuming constant mutation rate for all loci. With a Bayesian approach, a long chain with 20,000 genealogies to sample was run, with a sampling increment of 100 (thus, totaling 2,000,000 genealogies per replicate); the burn-in was set at 20,000. A static heating scheme was chosen (temperatures were specified to 1.00, 1.50, 3 and 1 × 10^6^), with uniform prior distribution both for Θ and *M* (min: 0; max: 500; delta: 50). The effective number of migrants per generation (*Nm*) among populations was estimated by using the formula 4*Nm* = Θ*M* (Beerli and Felsenstein, 2001). We could not estimate migration rate (*m*) values, because those determined from MIGRATE-N were mutation scaled (*M*), and mutation rates (μ) for microsatellites for the genus *Centaurea* were not available. Analyses were carried out at the CIPRES bioinformatic facility (Miller et al., 2010).

**FIGURE 2:**
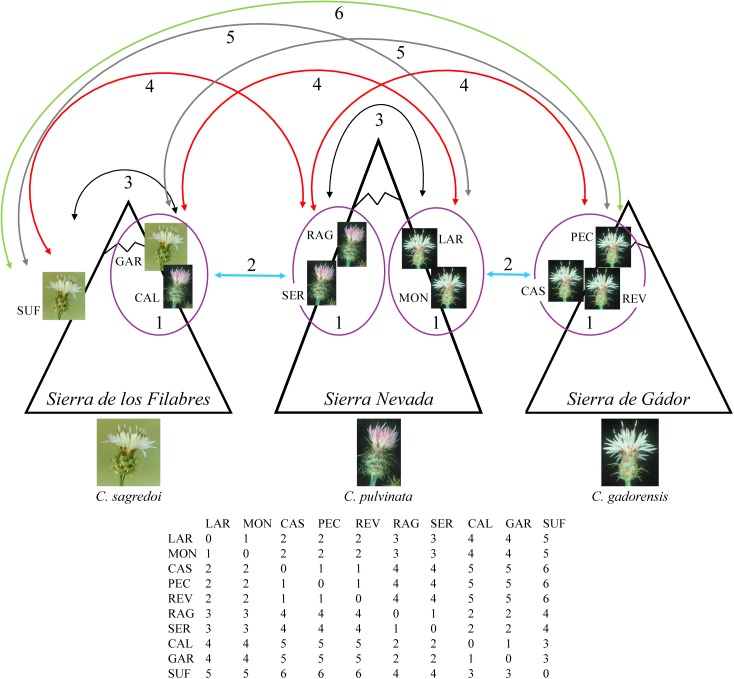
Diagram and matrix for the distance classes used in the spatial autocorrelation analysis. Class 1: intramountain-intramountainside (populations in the same mountain range, same mountainside; surrounded by a purple circle on the diagram and 1 in the matrix); class 2: intermountain-same valley (populations in two nearby mountains, on mountainsides facing the same valley; blue arrows on the diagram and 2 in the matrix); class 3: intramountain-intermountainside (populations in the same mountain range, different mountainsides; black arrows on the diagram and 3 in the matrix); class 4: intermountain-different valley (populations in two nearby mountains, on mountainsides facing different valleys; red arrows on the diagram and 4 in the matrix); class 5: one-mountain in between (populations in two mountains with one mountain in between; gray arrows on the diagram and 5 in the matrix); class 6: two-mountain in between (populations in two mountains with two mountains in between; green arrow on the diagram and 6 in the matrix).

Finally, we tested whether the observed genetic structure was a consequence of limited dispersal across space. Thus, we determined correlations between genetic differentiation [*F*_ST_ distances (Nei, 1987), determined with GENODIVE and transformed as *F*_ST_/(1 − *F*_ST_)] and geographical, climatic, and topographic factors by multiple matrix regression with randomization (MMRR; Wang, 2013). Geographical matrix included logarithms of geographical distances between populations. In order to generate the climatic and topographic distance matrices, we first compiled GIS data layers for 19 bioclimatic variables and a digital elevation model at 30 arc-sec resolution (ca. 1 km) from the WorldClim website ^1^ (Hijmans et al., 2005). Topographic variables were aspect (0° to 360° representing the azimuth that mountainsides are facing), elevation (in meters), and slope (in percentage). We extracted values for each variable at every locality using ARCGIS v10.2 (ESRI, Redlands, CA, United States), performed principal components analyses with the *prcomp* function and “scale = TRUE” in R, and calculated the distances between populations using the *dist* function in R. MMRR analysis was implemented using the *MMRR* function in R (Wang, 2013). Moreover, we also performed a MMRR analysis including a topographic resistance-based distance matrix and excluding direct geographical and topographic distances, to reflect interpopulation biological connectivity based on topography, and also a spatial autocorrelation analysis (using GENODIVE and the transformed *F*_ST_ distances). These analyses were used to test the genetic similarity between populations considering the topography and the orientation of the mountainsides on which the populations occur (Figure 2). Thus, if secondary contacts occurred in the lowlands during the glacial peaks and isolation at high altitudes during interglacial/postglacial periods, then we might expect that populations oriented to the same valley are genetically more closely related than those oriented toward different valleys. For resistance distance matrix, we generated a map of topographic suitability values (for aspect, elevation, and slope) using MAXENT v3.3 (Phillips et al., 2006), translated the suitability scores into resistance values (1-suitability) in order to generate a resistance layer, and then we formulated the distance matrix from the resistance layer by calculating pairwise least-cost path distances between populations using *gdistance* package in R. For the spatial autocorrelation analysis, the distance classes were (see Figure 2): intramountain-intramountainside (populations in the same mountain range, same mountainside; class 1), intermountain-same valley (populations in two nearby mountains, on the mountainside facing the same valley; class 2), intramountain-intermountainside (populations in the same mountain range, different mountainsides; class 3), intermountain-different valley (populations in two nearby mountains, on mountainsides facing different valleys; class 4), one-mountain in between (populations in two mountains with one mountain in between; class 5), two-mountain in between (populations in two mountains with two mountain in between; class 6). Significance of MMRR analyses and spatial autocorrelation was tested using 10,000 permutations.

### Ecological Niche Modeling (ENM)

The maximum entropy algorithm implemented in MAXENT was employed to evaluate the potential distribution of *C. gadorensis* for both the present and LGM climatic conditions. ENM was not performed with the other congeneric species studied (*C. pulvinata* and *C. sagredoi*) because the number of wild occurrences was not sufficient to establish reliable models (Pearson et al., 2007). Presence data for *C. gadorensis* were gathered from the Threatened Flora Information System (FAME) of the Andalusian regional government (Mateos et al., 2010; accessed under permission) and from the sampling sites of this study. After removing duplicate records within each pixel (30 arc-sec, ca. 1 km), 11 presence records resulted. To build the distribution model for the present climatic conditions, a set of 19 bioclimatic variables at 30 arc-sec resolution (ca. 1 km) covering the species distribution range and neighboring areas, and representative of the period 1950–2000, was downloaded from the WorldClim website. We performed a correlation analysis of the bioclimatic variables within the study area using SDMtoolbox v1.1b (Brown, 2014). From each pair or group of highly correlated variables (*r* ≥∣0.9∣), we selected the variable that contributed most to the model (attending to values of percent contribution and results of jackknife tests of variable importance) and had a clear response curve (i.e., those variables with flat or nearly flat response curves were not considered). The selected variables were isothermality (bio3), maximum temperature of the warmest month (bio5), minimum temperature of the coldest month (bio6), and precipitation seasonality (bio15). The distribution model under current conditions was then projected to the LGM using paleoclimatic layers simulated by the Community Climate System Model v4 (CCSM4; Gent et al., 2011) and the New Earth System Model of Max Planck Institute for Meteorology (MPI-ESM^2^). As these layers were available only at 2.5 arc-min resolution, they were interpolated to 30 arc-sec using the “Spline with Barriers” tool implemented in ARCGIS v10.2 (ESRI, Redlands, CA, United States).

Given the low number of occurrences for *C. gadorensis* (11), we used a methodology based on a jackknife (or ‘leave-one-out’) procedure to test the model (Pearson et al., 2007). Also, we used the Lowest Presence Threshold as the cut-off value to decide whether the discarded locality was ‘suitable’ or ‘unsuitable.’ The performance of the models was evaluated through success rate (percentage of right predictions) and statistical significance (see Pearson et al., 2007). To build the definitive models (i.e., using all occurrence points), we ran MAXENT 100 times using the bootstrap method. All ENM predictions were visualized in ARCGIS.

## Results

### Characteristics of the Molecular Markers Used

One of the microsatellite loci (the *13D10*) surveyed showed up to four alleles for many individuals of the three species. Since the diploid nature of some of these individuals was corroborated by visualizing their chromosomes in root meristematic cells from germinating seeds (following the protocol in Darlington and La Cour, 1969), the occurrence of individuals with up to four alleles (in *13D10* locus) was attributed to duplications of this locus within the genomes of the three species studied. Consequently, the locus *13D10* was excluded from the statistical analyses. There was no evidence of linkage disequilibrium for microsatellite loci (only loci *42CM27* and *CM15* showed linkage disequilibrium in the CAL population of *C. pulvinata*). With regard to the Hardy-Weinberg equilibrium (HWE), 14 of 60 population-by-locus tests deviated significantly, from which 10 corresponded to the *21D9* locus (in all populations). Null allele frequency was low for all combinations of loci and populations (mean < 0.05); only the bi-allelic locus *21D9* had a mean null allele frequency higher than 0.1 (mean = 0.157). Therefore, only null alleles in *21D9* appeared to explain the deviation from HWE. The alleles found and their observed frequencies for the different microsatellite loci, and null allele frequencies, are shown in the Supplementary Figure 2. The *trn*T–*trn*L sequence alignment was 559 base pairs in length, and it included five variable positions and seven indels.

### Genetic Diversity and Structure From Microsatellite Loci

In general, all three species showed very similar values of genetic-diversity indices (Table 1), with *C. pulvinata* and *C. sagredoi* showing the lowest value of variation in terms of heterozygosity (*H*_E_ = 0.65). At the population level, genetic-diversity values (*H*_E_) ranged from 0.54 of CAL (*C. pulvinata*) to 0.69 of REV (*C. gadorensis*), and those of allelic richness from 3.80 (LAR; *C. gadorensis*) to 5.75 (GAR and SUF; *C. sagredoi*) (Table 1). The comparison of DIC values for the full model (*f* > 0) and the null model (*f* = 0) resulted in inbreeding being significant in CAS, REV (*C. gadorensis*), RAG, CAL (*C. pulvinata*), and GAR (*C. sagredoi*); and also in *C. pulvinata* and *C. sagredoi* when the overall population estimation was made (see Table 1 and Supplementary Table 2). *Centaurea sagredoi* was the species with the highest number of private alleles (9), whereas *C. pulvinata* showed five and *C. gadorensis* three.

**Table 1:** Genetic variation in the populations of *C. gadorensis*, *C. pulvinata*, and *C. sagredoi* studied.

	*C. gadorensis*							*C. pulvinata*					*C. sagredoi*			
	LAR	MON	CAS	PEC	REV	Mean	OPE	RAG	SER	CAL	Mean	OPE	GAR	SUF	Mean	OPE
**Microsatellites**																
*A*	3.83	4.33	4.33	5.00	4.50	4.40	**6.83**	5.17	4.83	5.17	5.06	**7.17**	5.83	5.83	5.83	**7.67**
*Ar*	3.80	4.30	4.32	4.95	4.48	4.37	**6.09**	5.09	4.78	5.09	4.99	**6.71**	5.75	5.75	5.75	**7.66**
*Ap*	0	0	1	2	0	0.60	**3**	3	2	0	1.67	**5**	3	5	4.50	**9**
*H*_O_	0.63 (0.05)	0.55 (0.08)	0.59 (0.06)	0.62 (0.06)	0.68 (0.05)	0.61 (0.06)	**0.67 (0.03)**	0.61 (0.08)	0.59 (0.08)	0.52 (0.05)	0.57 (0.07)	**0.61 (0.05)**	0.56 (0.03)	0.62 (0.07)	0.59 (0.05)	**0.58 (0.06)**
*H_E_*	0.61 (0.04)	0.56 (0.05)	0.63 (0.04)	0.66 (0.03)	0.69 (0.03)	0.63 (0.04)	**0.70 (0.01)**	0.64 (0.04)	0.60 (0.05)	0.54 (0.05)	0.59 (0.05)	**0.65 (0.03)**	0.59 (0.02)	0.65 (0.04)	0.62 (0.03)	**0.65 (0.03)**
*f*	0.02 (0.02)	0.03 (0.02)	0.05^∗^ (0.04)	0.03 (0.03)	0.05^∗^ (0.03)	0.03 (0.03)	**0.03 (0.02)**	0.06^∗^ (0.04)	0.04 (0.04)	0.07^∗^ (0.05)	0.06 (0.04)	**0.06^∗^ (0.03)**	0.03^∗^ (0.02)	0.04 (0.03)	0.04 (0.03)	**0.07^∗^ (0.04)**
**cpDNA**																
*N° hap*	1	3	1	1	2	1.60	**6**	1	1	1	1	**3**	2	1	1.50	**3**
π	0.00	0.26 (0.19)	0.00	0.00	0.04 (0.06)	0.15	**1.26 (0.67)**	0.00	0.00	0.00	0.00	**0.76 (0.43)**	0.20 (0.16)	0.00	0.10	**15.3 (7.69)**
*H*	0.00	0.38 (0.18)	0.00	0.00	0.20 (0.15)	0.12	**0.70 (0.03)**	0.00	0.00	0.00	0.00	**0.69 (0.02)**	0.53 (0.09)	0.00	0.27	**0.65 (0.065)**

*A, mean number of alleles per locus; Ar, Allelic richness per population and species based on minimum sample size of 28 individuals (60 individuals for specific level); Ap, number of private alleles; H_O_, observed heterozygosity (corrected for null alleles); H_E_, expected heterozygosity (corrected for null alleles); F_IS_, inbreeding coefficient (accounting for null alleles); N° hap, number of haplotypes; H, haplotype diversity; π, nucleotide diversity (×10^−2^). ^∗^Inbreeding supported by DIC comparison. In bold, values for overall populations estimation (OPE).*

AMOVA analysis showed that most of the genetic variation occurred within populations (85%), and that the genetic differentiation between species was lower (*F*_CT_ = 0.046, *P* = 0.001) than the genetic structuring between populations within species (*F*_SC_ = 0.105, *P* < 0.001; Table 2). Pairwise *F*_ST_ comparison showed higher differentiation between *C. gadorensis* and *C. sagredoi*, and between *C. gadorensi*s and *C. pulvinata*, than between *C. pulvinata* and *C. sagredoi* (Supplementary Table 3). At the population level, all pairwise comparisons were significant (Supplementary Table 4).

**Table 2:** Hierarchical analysis of molecular variance (AMOVA).

Source of variation	d.f.	Sum of squares	Variance components	Percentage of variation	Fixation indices	*P*-value
***Microsatellites***						
Among species	2	62.549	0.0936	4.63	*F*_CT_ = 0.046	0.001
Among populations within species	7	96.056	0.2023	10.01	*F*_SC_ = 0.105	<0.001
Within populations	590	1017.667	1.7249	85.36	*F*_ST_ = 0.146	<0.001
Total	599	1177.272	2.0208			
***cpDNA***						
Among species	2	694.360	6.5641	31.18	*F*_CT_ = 0.312	0.045
Among populations within species	7	1005.840	14.3558	68.19	*F*_SC_ = 0.991	<0.001
Within populations	90	12.000	0.1333	0.63	*F*_ST_ = 0.994	<0.001
Total	99	1712.200	21.0533			

The Bayesian clustering method, as implemented in STRUCTURE, recognized two genetic clusters as the uppermost hierarchical level of genetic partitioning according to the highest Δ*K* peak (Figure 1A and Supplementary Figure 3). These clusters could be identified as *C. gadorensis* and *C. sagredoi*. Meanwhile, *Centaurea pulvinata* showed a clear admixture of the *C. gadorensis* and *C. sagredoi* genomes, although the contribution of each species was different, depending on the *C. pulvinata* population. Thus, individuals of the SER population were almost equally assigned to both clusters (*Q* = 0.55, *C. gadorensis* cluster vs. *Q* = 0.45, *C. sagredoi* cluster), whereas individuals of the RAG and CAL populations were clearly assigned to the *C. sagredoi* cluster (*Q* = 0.84 and *Q* = 0.89, respectively). The results interpreted using the method of Puechmaille (2016) revealed nine clusters (*K* = 9) as the most likely group structure (Supplementary Figure 3), considering locations with *Q* ≥ 0.5 in any inferred cluster. This value was also recovered by the mean posterior probabilities lnP(*K*) and it coincided with a second highest Δ*K* peak (Supplementary Figure 3). The mean membership coefficient per population ranged from 0.508 to 0.760 for individuals across the nine inferred clusters. In this case, most of individuals comprising each cluster were from one sampling location, with two exceptions, REV, RAG, and PEC populations. REV population (*C. gadorensis*) presented a mixture of individuals belonging to differentiated genetic clusters [the clusters that are approximately assigned to the CAS, MON (*C. gadorensis*) and SER (*C. pulvinata*) populations]. The RAG population (*C. pulvinata*) showed a clearly mixed ancestry involving the genetic cluster for GAR (*C. sagredoi*). PEC population (*C. gadorensis*) was the only population with *Q* < 0.5 in any inferred cluster and, therefore, could not be assigned to a specific cluster. All clusterings, from *K* = 2 to *K* = 11, are represented in the Supplementary Figure 3.

**FIGURE 3:**
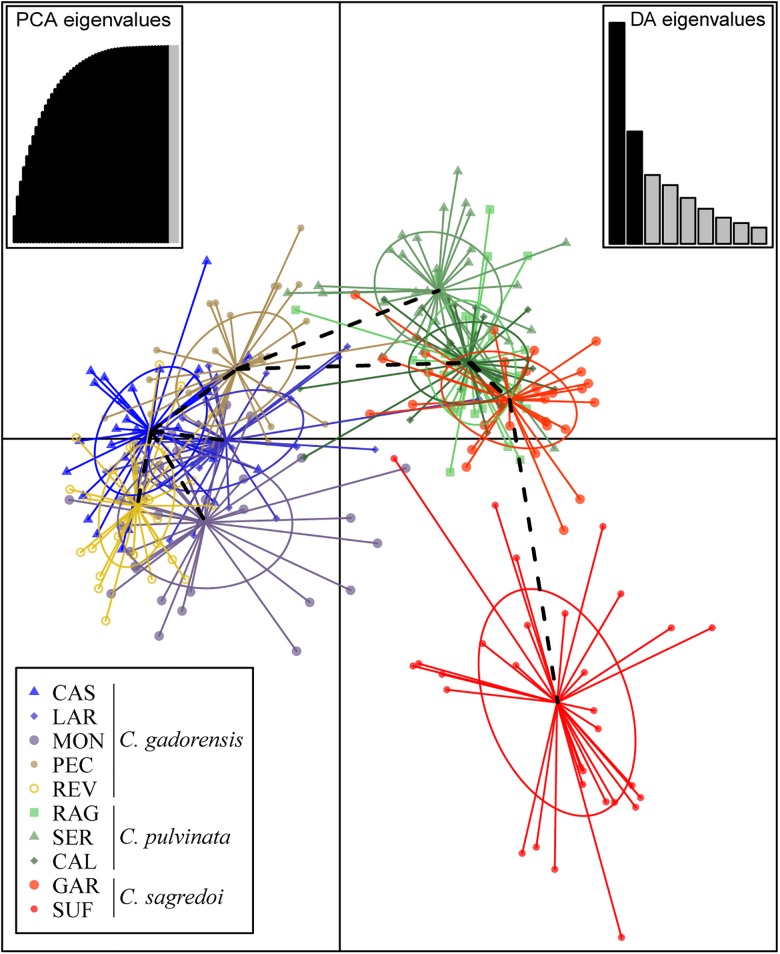
Result of the discriminant analysis of principal components (DAPC) using microsatellites. For population codes, see Supplementary Table 1.

Discriminant analysis of principal component clustering showed that the first discriminant component separated populations of *C. gadorensis* from the other two species, and the second discriminant component separated the SUF population of *C. sagredoi* from *C. pulvinata* populations and GAR of *C. sagredoi*, which was intermingled with *C. pulvinata* populations (Figure 3). BARRIER software detected sharp genetic breaks that coincided mostly with the ridgelines of both Sierra Nevada and Sierra de los Filabres (i.e., there are putative genetic barriers between populations located on the northern and southern mountainsides of these two mountain ranges, especially notable after the sixth barrier was added; Supplementary Figure 4); putative barriers detected between neighboring populations in the same mountainside and between populations on nearby mountain ranges oriented toward the same valley proved less significant (see Supplementary Figure 4).

### Diversity and Structure of cpDNA Haplotypes

For the cpDNA (Figure 1A,B and Table 1), we found 10 haplotypes in total. At the species level, *C. gadorensis* harbored six haplotypes, and *C. pulvinata* and *C. sagredoi* three each. Haplotypes were not segregated taxonomically (at the species level) or geographically (according to the mountain range), but rather mainly by population (Figure 1A,B). The main haplotype (H1), central in the network (Figure 1B), was shared by populations of all three species, the only haplotype being found in PEC (*C. gadorensis*) and SER (*C. pulvinata*), and the most frequent in GAR (6 of 10 sequences, *C. sagredoi*) and MON (8 of 10 sequences, *C. gadorensis*). Only one other haplotype (H2) was found in more than one population, whereas the remaining haplotypes were exclusive to single populations (and, in many cases, with all their individuals fixed to that haplotype; Figure 1A,B).

According to the AMOVA results (Table 2), most variation was found in the interpopulation component (68%) due to the strong intrapopulation uniformity of the haplotype variants (*F*_ST_ = 0.994, *P* < 0.001) and the very high interpopulation differentiation (*F*_SC_ = 0.991, *P* < 0.001). The species are not clearly differentiated with the chloroplastidial marker, where the coefficient of fixation between species was much lower than that between populations and was at the limit of significance (*F*_CT_ = 0.31, *P* = 0.045; Table 2). The interspecific pairwise *F*_ST_ showed the highest differentiation in those of *C. sagredoi* with regard to *C. gadorensis* and *C. pulvinata* (Supplementary Table 3).

The Bayesian clustering based on cpDNA data revealed seven clusters (Figure 1C). One cluster contained the H1 sequences (from MON, PEC, SER, and GAR); other included haplotypes H2 (from CAS and REV) and H3 (from REV); haplotypes H4 (from LAR) and H8 (from CAL) were merged into another cluster; H5 and H6 (both from MON) formed another cluster; while sequences from RAG (H7), SUF (H10), and four sequences from GAR (H9) formed the remaining three clusters (Figure 1C).

### Gene Flow and Spatial Correlation

The results of BAYESASS clearly indicated no current exchange of genes (with the relative exception of REV–CAS; Supplementary Table 5). By contrast, considerable values of gene flow were recovered with MIGRATE-N for all pairwise comparisons [with a mean value of *Nm* = 1.466, above the cutoff value of Wright (*Nm* = 1; Wright, 1931); Supplementary Table 6]. According to 2MOD analyses, the gene-flow model was significantly more favored than a pure drift model both at the species and at the sampling locality levels (*P* = 1, BF = 100,000; *P* = 0.99, BF = 20,000; respectively).

MMRR analysis revealed significant contribution of the isolation by distance to the genetic differentiation of the populations (β = 0.035, *P* = 0.017), but did not identify any contribution of the climatic or topographic variables (Table 3). However, a more detailed examination showed that this model was influenced by *C. gadorensis*, and specifically by the PEC and REV populations: when they were eliminated, there was no effect of isolation by distance (β = 0.011, *P* = 0.643). On the other hand, when we tested the effect that the mountainside orientation of populations exerted on genetic differentiation, the MMRR analysis identified a significant contribution (using the resistance matrix for the topographic variables; Table 3). Similarly, the spatial autocorrelation analysis showed a significant genetic similarity between neighboring populations in the same mountain mountainside (class 1: intramountain-intramountainside; *r* = 0.315, *P* = 0.006), and between populations on nearby mountain ranges oriented toward the same valley (class 2: intermountain-same valley; *r* = 0.366, *P* = 0.004). On the contrary, there was no significant genetic similarity among other distance classes, including intramountain range populations on mountainsides with different orientations (class 3; Figure 2) (Table 3).

### Ecological Niche Modeling

**FIGURE 4:**
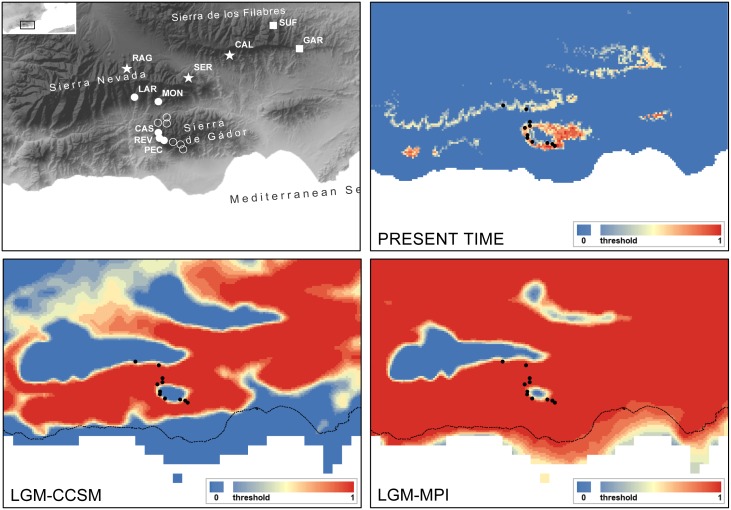
Potential distribution of *Centaurea gadorensis* drawn with MAXENT. Top left, topographic map showing the location of genetically studied populations of *C. gadorensis* (full circles), *C. pulvinata* (stars) and *C. sagredoi* (squares). Additional presence records of *C. gadorensis* used for ecological niche modeling (ENM) are shown in empty circles. Top right, at the present time; bottom left, at the Last Glacial Maximum (LGM, ca. 21,000 years BP) using the Community Climate System Model (CCSM); bottom right, at the LGM using the Model of Max Planck Institute for Meteorology (MPI-ESM). Black dots indicate current populations of the species, whereas the dashed line shows the present coastline. The probability of presence is shown as continuous values from the threshold (defined as Maximum Sensitivity plus Specificity) to 1. This figure has been generated with ARCGIS.

The model for *C. gadorensis* performed reasonably well, as we found a high success rate (0.818) and statistical significance (*P* < 0.001) in the jackknife test. For the present time frame, several middle-elevation (1,000*–*2,000 m) mountain areas within the study area appear to be suitable for *C. gadorensis*. Such areas included peripheral mountains of both Sierra Nevada and Sierra de los Filabres, most of Sierra de Gádor (with the exception of its mountaintops), and other small mountain ranges. By contrast, the intermountain valleys, usually below 1,000 m, appeared to be unsuitable (Figure 4). Projections of the species niche to the LGM were considerably different, showing all the lowlands (intermountain valleys) and midlands usually up to 1,600*–*1,800 m (Figure 4) to be suitable, thus suggesting a clear scenario of population connectivity. The LGM projections should be interpreted, however, with extreme caution given the uncertainty of projections for the past using a small number of occurrences (11).

**Table 3:** Results of the spatial correlation analyses.

	MMRR				Mountainside orientation^1^			
	All populations		Without PEC, REV		MMRR		Spatial autocorrelation	
	β	*P*	β	*P*	β	*P*	*r*	*P*^2^
Cd	−0.002	0.580	0.005	0.450	−0.005	0.270	–	–
Gd	**0.035**	**0.017**	0.011	0.642	–	–	–	–
Td	−0.007	0.601	−0.017	0.418	**0.5 × 10^−3^**	**0.013**	–	–
dc1	–	–	–	–	–	–	**0.315**	**0.006**
dc2	–	–	–	–	–	–	**0.366**	**0.004**
dc3	–	–	–	–	–	–	−0.130	0.190
dc4	–	–	–	–	–	–	−0.130	0.168
dc5	–	–	–	–	–	–	−0.317	0.022
dc6	–	–	–	–	–	–	−0.148	0.191

*Cd, climatic distances; dc, distance class (1–6; see the section “Materials and Methods”); Gd, geographical distances; Td, topographic distances. ^1^ Analyses performed to test the effect of the orientation of the mountainsides inhabited by the populations; here Td correspond to the topography-based resistance distances. ^2^ Significant P-values at the 5% nominal level after sequential Bonferroni correction. All significant values are shown in bold.*

## Discussion

The subsection *Willkommia* of sect. *Centaurea* has been considered an example of a group of species that had diverged following a schizo-endemic pattern (Blanca, 1981a), in which fragmentation and isolation of a widespread ancestral taxon favored the allopatric differentiation of closely related range-restricted endemic species sharing the same chromosome number. Nevertheless, molecular systematic studies on the subsection (Suárez-Santiago et al., 2007b) and the section *Centaurea* (Hilpold et al., 2014b), as well as the evolutionary analysis of a satellite-DNA family within the subsection (Suárez-Santiago et al., 2007c) clearly indicate that diversification of *Willkommia* has followed a model of reticulate evolution.

### Secondary Contacts and Historical Gene Flow Between *C. gadorensis*, *C. pulvinata*, and *C. sagredoi* Populations

The BAYESASS analysis indicated almost no current gene flow between the populations studied (Supplementary Table 5, except for CAS and REV), this being an expected result given that all these populations have a high degree of geographical isolation, except populations CAS and REV of *C. gadorensis* in the Sierra de Gádor (Figure 1A). By contrast, our data support the occurrence of historical gene flow among the species studied, as shown by the results of the demographic analyses conducted with MIGRATE-N (Supplementary Table 6) and 2MOD. In fact, the historical gene-flow rates (including the interspecific rates) should be regarded as high (range *Nm* = 1.138*–*2.013, mean = 1.466; Supplementary Table 6), particularly if we compare them with other congeneric species and partly using the same microsatellite loci. *Nm* values for populations studied here are much higher than those reported for other endemic *Centaurea* species of the subsect. *Phalolepis*, phylogenetically close to *Willkommia* (Garcia-Jacas et al., 2006; Suárez-Santiago et al., 2007b) with disjunct areas in Greece (mean *Nm* = 0.534; López-Vinyallonga et al., 2015), Italy (mean *Nm* = 0.645; Garcia-Jacas et al., 2019), and Turkey (mean *Nm* = 0.466; López-Pujol et al., 2016). The high levels of historical gene flow detected in the present study are consistent with the low genetic differentiation observed between species (Table 2 and Supplementary Table 3), which are significant but much lower than those reported for both Greek, Italian, and Turkish *Centaurea* subsect. *Phalolepis* (*F*_ST_ = 0.243, 0.232, and 0.198, respectively; López-Vinyallonga et al., 2015; López-Pujol et al., 2016; Garcia-Jacas et al., 2019). Moreover, we have not detected isolation by distance (except among *C. gadorensis* populations of Sierra de Gádor), suggesting that the lineage distribution is the result of historical phylogeography. All this evidence, together with the genetic admixture found within the morphologically well-characterized populations (Figure 1A and Supplementary Figure 3) supports the hypothesis of secondary contacts between populations of differentiated species. Although we were not able to ascertain exactly the time when the inferred gene flow with MIGRATE-N took place [as mutation rates (μ) for microsatellites for the genus *Centaurea* are unknown], this would have occurred around the LGM (we found a time interval of 1,100*–*46,000 years ago, following the approximation of López-Vinyallonga et al., 2015).

According to our results *C. pulvinata* must have been a major player in the hybridization and introgression events, hybridizing with both *C. gadorensis* and *C. sagredoi*. However, we detected no clear evidence of hybridization between *C. gadorensis* and *C. sagredoi*. The values of genetic differentiation (Supplementary Table 3) and the results of the genetic structure analyses (Figure 1A, 3 and Supplementary Figure 3) show that *C. gadorensis* is highly differentiated from *C. sagredoi*. Instead, these same analyses show a close relationship between *C. pulvinata* and *C. sagredoi* with microsatellite markers, suggesting the same nuclear genetic background between the two. This relationship is consistent with the phylogenetic analysis of *Willkommia* (Suárez-Santiago et al., 2007b), where both species belong to the clade called Nevado-Filábride as sister species. The close genetic relationship between *C. pulvinata* and *C. sagredoi* based on microsatellites also agrees with morphological data, which have traditionally related the two species (Blanca, 1980, 1981b, 1984), and furthermore agrees with ecological data, as the species have the same requirements in terms of soil type (acidic soils derived from schist; Blanca, 1980, 1981b, 1984). The large proportion of individuals with mixed ancestry detected in one of the two *C. sagredoi* populations (GAR) and the almost absence in the geographically isolated population SUF (Figure 1A) at *K* = 3 (Supplementary Figure 3) support introgression from *C. pulvinata* into *C. sagredoi* (see also Figure 3). We have also detected hybridization and introgression between *C. gadorensis* and *C. pulvinata* despite their not being as close as the pair *C. pulvinata*/*C. sagredoi*, (Figure 1A). Indeed, in *Centaurea* hybridizations have been found even between different sections (e.g., Hilpold et al., 2014b).

### Influence of the Topographic Features on the Reticulate Evolution Pattern

Mountains are among the main topographic factors influencing the genetic structure of plant species. It is widely accepted that during glacial/interglacial cycles of the Pleistocene the wide diversity of microhabitats in the Mediterranean mountains allowed species to migrate along altitudinal gradients, favoring differentiation of isolated populations (Hewitt, 1999; Gómez and Lunt, 2007; Médail and Diadema, 2009; Jiménez-Mejías et al., 2015). Also, hybridization and reticulate evolution of previously isolated lineages resulted in complex evolutionary patterns (cf. Thompson, 2005; Nieto Feliner, 2011, 2014), as has been described for the *Willkommia* subsection (Suárez-Santiago et al., 2007b).

Here, we highlight the way in which the orientation of the mountainsides inhabited by the populations enabled secondary contacts between populations during glacial peaks. Thus, we suggest that the intermountain valleys are the areas where secondary contacts occurred, and that the probability of these secondary contacts was determined by the orientation of the populations toward the same valley or toward different valleys. Our data show that current populations harbor genetic signatures of this model, so that the observed genetic differentiation is explained, in part (isolation is also involved; see below), by the effect of their orientation. This finding supports that they are the result of migration at high altitudes from contact areas and not of remaining *in situ* during the glacial/interglacial cycles. The main evidence for the effect of the topography and orientation of the populations on the observed genetic differentiation comes from the spatial correlation analyses, which resulted in populations being significantly related on the same mountainside or different mountainsides but oriented toward the same valley (Table 3), regardless of their taxonomy. The other evidence comes from the analysis with BARRIER (Supplementary Figure 4), which identified the mountain ridgelines of Sierra Nevada and Sierra de los Filabres as barriers to the gene flow between populations on both sides of each mountain range. Given the lack of isolation by distance and the patterns of historical/contemporary gene flow, these data, combined with species-distribution modeling (Figure 4), support a scenario in which allopatric populations were forced by the Quaternary glacial periods to expand their ranges at lower altitudes, coming into contact and hybridizing in the intermountain valleys. Meanwhile, during the interglacial/postglacial (warm) periods, populations would retreat again to higher elevations on the mountains (as occurs today), starting a period of isolation and differentiation.

The geographical distribution of the populations of *C. sagredoi* and *C. pulvinata* (Figure 1A), together with the genetic pattern detected with the microsatellites for both species (Figure 1A, 3, Supplementary Figure 3, and Supplementary Tables 3, 6), fit the hypothesis of altitudinal migrations following the pattern of climatic oscillations and recurrent genetic exchanges. Populations of the southern mountainside of Sierra de los Filabres and the northern mountainside of Sierra Nevada would have formed a zone of genetic contact in the valley located between the two mountains (Figure 1A, 2). A similar example has been described for the genus *Armeria* in Sierra Nevada (Fuertes Aguilar et al., 2011). In this case, contact between the parental species of the hybrid *A. filicaulis* subsp. *nevadensis*, namely *A. splendens* and *A. filicaulis*, is explained by the lowering of the altitudinal range of *A. splendens* driven by the contraction of vegetation belts during the LGM. The high isolation of SUF (*C. sagredoi*) on the northern mountainside of Sierra de los Filabres (Figure 1A) is the likely the reason for its greater genetic differentiation [Figure 3, Supplementary Figure 3 (with its own cluster from *K* = 3), and Table 1 (with a large number of private alleles)]. During the glacial periods, secondary contact with other populations of *C. sagredoi* (GAR) or with *C. pulvinata* would have been rather unlikely.

As in the case between the southern mountainside of Sierra de los Filabres and the northern mountainside of Sierra Nevada, there is a clear genetic connectivity between populations located at the southern mountainside of Sierra Nevada and the northern mountainside of Sierra de Gádor, all belonging to *C. gadorensis* (Figure 1A, 3 and Supplementary Figure 3). Although such genetic connectivity is expected for conspecific populations, it is also attributable to the glacial periods due to the lack of isolation by distance, when plants would have descended to lowlands and exchanged genes. Such a scenario is also reflected with the ENM, which unambiguously indicates that areas of presumed genetic exchange would have occupied extensive intermountain valleys during the LGM (Figure 4). On the contrary, during the interglacial periods such as the present time, plants would have moved upward, constraining the exchange of genes, as indicated by the BAYESASS analysis (Supplementary Table 5).

Within *C. gadorensis*, it is somewhat surprising to find genetic differences between LAR and MON populations despite the general pattern of genetic similarity between neighboring populations on the same mountainside (see Supplementary Figure 3 for *K* = 9, Supplementary Table 4). In the STRUCTURE analysis, population LAR shows its own cluster from *K* = 7 (Supplementary Figure 3), and there is a presumed genetic barrier between LAR and MON (Supplementary Figure 4). Both populations show the highest pairwise *F*_ST_ value within the species, and LAR harbors the H4 haplotype while MON shows mainly H1 (Figure 1A–C). Such genetic differences might be due to topography, as both populations are separated by two subsidiary ridges that reach 1,800 m at the latitude of LAR. An alternative or complementary explanation may be demography: LAR and MON are two small populations with 1,197 individuals pooling the two populations (Giménez et al., 2011). Genetic drift would have been severe in these small localities, leading to the present patterns of genetic differentiation in contrast to the populations in Sierra de Gádor, with a combined estimated size of 33,648 individuals (Giménez et al., 2011).

On the other hand, our results also show the genetic signature left by population-divergence processes in allopatry. Population isolation, as a consequence of fragmentation processes and/or bottlenecks/founder events, generally results in populations with especially low levels of genetic diversity in the cpDNA (as result of the smaller effective size of the cpDNA compared to the nuclear genome; e.g., Bittkau and Comes, 2005), as well as in the absence of isolation by distance between populations (Hutchison and Templeton, 1999). We have detected both situations (Tables 1, 3 and Figure 1A–C), which, together with the second hierarchical level of genetic structure observed for microsatellites (*K* = 9, Supplementary Figure 3) and AMOVA results (Table 2), points to the impact of genetic drift on the populations studied, although other factors cannot be ruled out (e.g., selection). Given the evidence of secondary contacts already discussed, genetic drift is probably due to the founding events during the retraction in altitude of the populations from the contact sites during the interglacial periods. Previous studies of secondary contact and hybridization during glacial periods and isolation in warm interglacials show a clear geographical structure (e.g., Albaladejo et al., 2005; Maguilla and Escudero, 2016; Schneeweiss et al., 2017). By contrast, our results do not show a clear geographical trend through the sharing of haplotypes among taxa in different geographical areas; nor do they show a taxonomic pattern (Figure 1A,C). This evidence points to the recurrence of secondary contacts and possible sorting of plastid lineages during the population retraction after secondary contacts, coupled with genetic drift.

### High Genetic Diversity in the Species of the Subsection *Willkommia* of *Centaurea*: An Expected Outcome for Plant Species From a Mediterranean Mountain Refuge

Genetic diversity detected with microsatellites within the species studied is substantial (mean *H*_E_ for the 10 populations studied = 0.616; Table 1), higher than expected for endemic species in general (*H*_E_ = 0.420; Nybom, 2004), and comparable to expected rates for widespread species (*H*_E_ = 0.620; Nybom, 2004). Similar levels of genetic variability were found in species of *Centaurea* subsect. *Phalolepis* using almost the same set of microsatellite loci (mean *H*_E_ = 0.587 and 0.504 for the Greek and Anatolian taxa, respectively; López-Vinyallonga et al., 2015; López-Pujol et al., 2016). For narrow endemic plants, the expected levels of genetic diversity are low, but in the Mediterranean mountains the endemisms appear to harbor moderate to high levels (cf. Jiménez-Mejías et al., 2015) such as those found previously in *Centaurea* subsect. *Phalolepis* and now in the subsect. *Willkommia*. The reason for such polymorphism in Greece and Turkey was the occurrence of populations within mountainous, environmentally stable refugia, and the same reason may also account for our present results, given that all three species are distributed within the Sierra Nevada/Gata refuge (Médail and Diadema, 2009). It is widely agreed, both on the basis of paleoecological and genetic data, that mountainous habitats in the main Mediterranean peninsulas (Iberian, Italian, and Balkan, but also Anatolian) have acted as large refugia both for plants and animals (Bennett et al., 1991; Hewitt, 1996, 1999; Taberlet et al., 1998; Gómez and Lunt, 2007). Endemisms and phylogeographic hotspots are often linked to climatic stability and topographic heterogeneity (Carnaval et al., 2009; Médail and Diadema, 2009; López-Pujol et al., 2011; Harrison and Noss, 2017). In addition to the occurrence within a refugium, hybridization and an increased effective population size might have influenced the levels of genetic diversity of these three *Centaurea* species by increasing it during the secondary contacts (Frey et al., 2012; Seehausen et al., 2014).

## Author Contributions

SB-MS and JL-P performed the laboratory work. VS-S, JL-P, SB-MS, and RV analyzed the data. NG-J and VS-S conceived and designed the studies. GB and RV participated in the design. VS-S drafted the manuscript and all the authors participated in the editing of the manuscript. All the authors read and approved the final manuscript.

## Conflict of Interest Statement

The authors declare that the research was conducted in the absence of any commercial or financial relationships that could be construed as a potential conflict of interest.

## Footnotes

1 www.worldclim.org

2 http://www.mpimet.mpg.de/en/science/models/mpi-esm/
