# Supplementary material for: Influence of the Quaternary Glacial Cycles and the Mountains on the Reticulations in the Subsection Willkommia of the Genus Centaurea
Source: Front Plant Sci. 2019 Mar 21;10:303. doi: 10.3389/fpls.2019.00303 (PMC6437100; doi:10.3389/fpls.2019.00303)
Supplement: Supplementary file 1 [file Data_Sheet_1.docx]

Supplementary Material

**Influence of the Quaternary Glacial Cycles and the Mountains on the Reticulations in the Subsection *Willkommia* of the Genus *Centaurea***

*Samira Ben-Menni Schuler^1^, Jordi López-Pujol^2^, Gabriel Blanca^1^, Roser Vilatersana^2^, Núria Garcia-Jacas^2^ and Víctor N. Suárez-Santiago^1*^*

*** Correspondence:** Víctor N. Suárez-Santiago: vsuarez@ugr.es

# Supplementary Figures and Tables

## Supplementary Figures


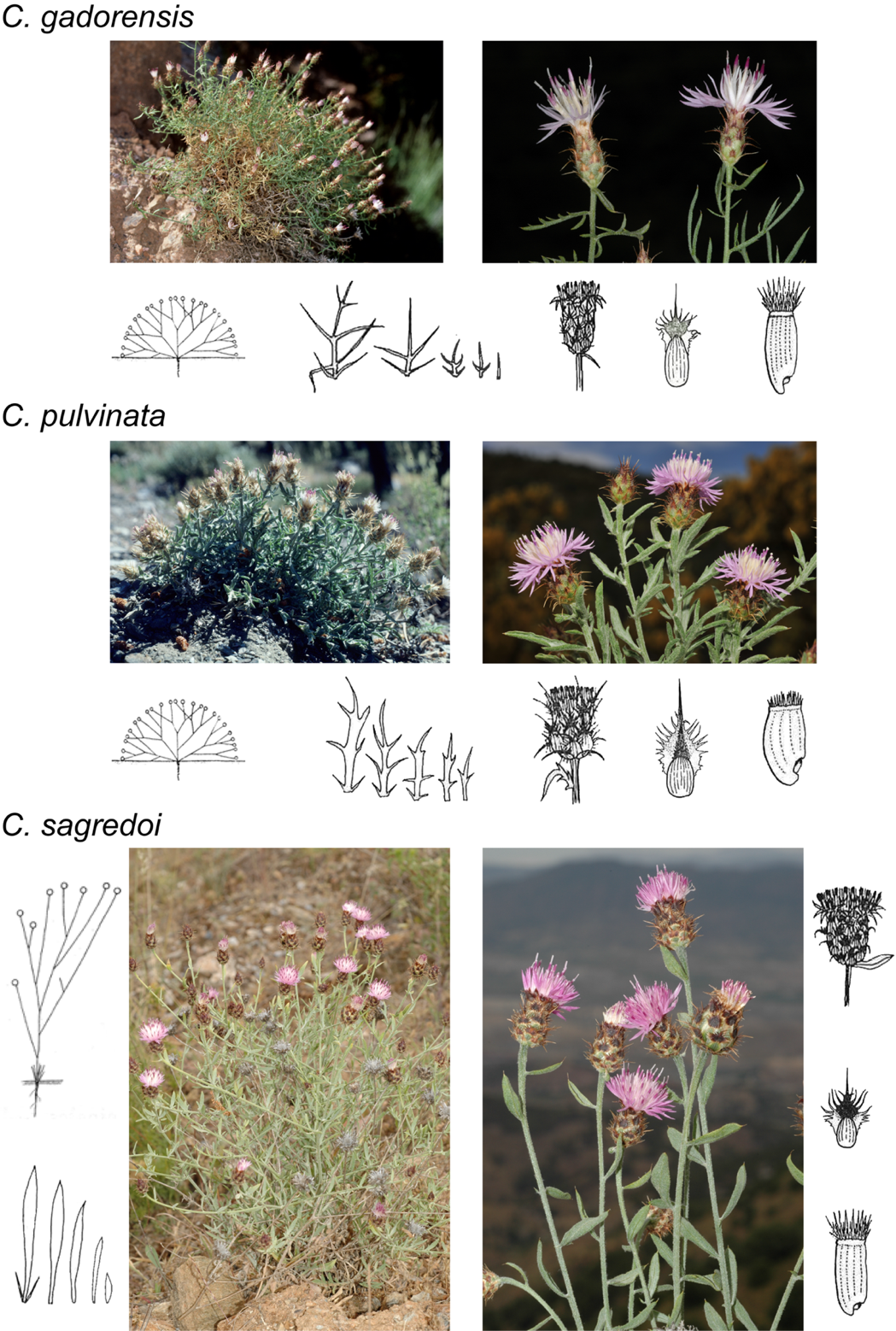


**Supplementary Figure 1.** Photographs and icons showing the main differential features of the three species studied: habit type, upper leaves, capitula, middle phyllaries, and achenes. *Centaurea gadorensis* has a pulvinular habit, with stems and branches intricately arranged; upper leaves pinnatisect, with 1–5 pairs of segments, sometimes the closest to the capitula linear and entire; involucre narrowly cylindrical to ovoid-subcylindrical with attenuated base; the terminal spine of the middle phyllaries 3–4 mm in length; and a pappus 1–2 mm long. *Centaurea pulvinata* has also a pulvinular habit, with stems and branches intricately arranged; upper leaves pinnatisect, with 1–3 pairs of segments; involucre ovoid to ovoid-subglobose with rounded base; the terminal spine of the middle phyllaries (3)4–7(8) mm in length; and a pappus 0.7(1) mm long. Finally, *C. sagredoi* has not pulvinular habit, with stems and branches loosely arranged; upper leaves entire, linear to linear-oblanceolate; involucre ovoid to ovoid-subglobose with rounded base; the terminal spine of the middle phyllaries (2)2.5–3.5(4) mm in length; and pappus 1–2 mm long. Photographs and drawings made by G. Blanca.

**(A)**

**(B)**

**Supplementary Figure 2.** (A) Bar plot showing the distribution among species of the allele frequencies for microsatellite loci. Locus “0”: null allele. *: Private allele. (B) Bar plot showing the population null allele frequencies.

##

**Supplementary Figure 3.** Bar plots showing the STRUCTURE assignment test for *K* = 2 to *K* = 11, using microsatellite data and assuming the admixture model. Delta *K* method (Evanno et al., 2005) was used to identify the uppermost hierarchical level of genetic structure (*K* = 2), while lnP(K) (Pritchard et al., 2000) and MedMedK, MedMeaK, MaxMedK, and MaxMeaK (with membership coefficient threshold of 0.5; Puechmaille, 2016) were used to identify other levels of genetic partitioning (*K* = 9). The *K* values selected by each estimator are shown on the right. For population codes, see Supplementary Table 1.

**Supplementary Figure 4.** BARRIER analysis with one, three, and six barriers, with significance tested by 1,000 bootstrap matrices of Nei’s genetic distance *D*_a_ (Nei et al., 1983). Numbers indicate bootstrap support (after 1, 3, and 6 barriers).

## Supplementary Tables

**Supplementary Table 1.** Sampling details of *Centaurea* populations used in the present study.

| Population code | Location | Voucher | Geographical coordinates (N/W) | Altitude  (m a.s.l.) |
| --- | --- | --- | --- | --- |
| *C. gadorensis* |  |  |  |  |
| LAR | Sierra Nevada (Granada), Laroles | GDAC 16542 | 37º02’20’’/3º00’28’’ | 1,450 |
| MON | Sierra Nevada (Almería), Monterrey | GDA 46420 | 37º01’20’’/2º54’13’’ | 1,380 |
| CAS | Sierra de Gádor (Almería), Minas de Castala | GDAC 6789 | 36º54’20’’/2º53’54’’ | 1,480 |
| PEC | Sierra de Gádor (Almería), Pecho Cuchillo | HUAL 4930-1 | 36° 51' 57”/2° 51' 55” | 1,925 |
| REV | Sierra de Gádor (Almería), El Reventoncillo | HUAL 20367 | 36º53’34’’/2º53’56’’ | 1,327 |
| *C. pulvinata* |  |  |  |  |
| RAG | Sierra Nevada (Granada), Puerto de la Ragua | GDAC 6020 | 37º08’33’’/3º02’27’’ | 1,580 |
| SER | Sierra Nevada (Almería), Venta del Serbal | BC 942035 | 37º06’32’’/2º46’15’’ | 1,367 |
| CAL | Sierra de los Filabres (Almería), Calar Alto | BC 947401 | 37º11’24’’/2º35’24’’ | 1,700 |
| *C. sagredoi* |  |  |  |  |
| GAR | Sierra de los Filabres (Almería), Collado García | GDAC 6018 | 37º12’37’’/2º17’00’’ | 1,269 |
| SUF | Sierra de los Filabres (Almería), Suflí | GDAC 6014 | 37º19’15’’/2º22’29’’ | 1,034 |

BC, herbarium of the Botanical Institute of Barcelona; GDA/GDAC, herbarium of the University of Granada; HUAL, herbarium of the University of Almería.

**Supplementary Table 2.** Average inbreeding coefficient (*f*) estimates based on microsatellite markers for the study populations and species, using the software INEST. 95% credibility interval around *f* is shown [HPDl(95%), HPDl(95%)]. DIC, Deviance Information Criterion measuring the overall fit of the full model (*f* > 0) and null model (*f* = 0) to the data. *: significant *f*  by comparison of the DIC values.

| Population | *f* | HPDl(95%) | HPDh(95%) | DIC (*f* > 0) | DIC (*f* = 0) |
| --- | --- | --- | --- | --- | --- |
| LAR | 0.0196 | 0 | 0.0619 | 623.283 | 618.137 |
| MON | 0.0267 | 0 | 0.0747 | 593.259 | 591.627 |
| CAS | 0.0504* | 0 | 0.1299 | 728.882 | 729.595 |
| PEC | 0.0304 | 0 | 0.0869 | 770.668 | 768.973 |
| REV | 0.0498* | 0.0003 | 0.1145 | 756.349 | 757.49 |
| RAG | 0.0574* | 0.0001 | 0.1386 | 707.813 | 709.947 |
| SER | 0.0414 | 0 | 0.1135 | 680.283 | 679.968 |
| CAL | 0.0701* | 0.0011 | 0.1771 | 635.352 | 635.901 |
| GAR | 0.0332* | 0 | 0.082 | 780.019 | 781.409 |
| SUF | 0.0401 | 0.0001 | 0.1087 | 787.603 | 787.115 |

| Species | *f* | HPDl(95%) | HPDh(95%) | DIC (*f* > 0) | DIC (*f* = 0) |
| --- | --- | --- | --- | --- | --- |
| *C. gadorensis* | 0.0333 | 0.0001 | 0.0751 | 4047.068 | 4046.904 |
| *C. pulvinata* | 0.0627* | 0.0021 | 0.1291 | 2335.508 | 2340.557 |
| *C. sagredoi* | 0.0707* | 0.0031 | 0.1477 | 1765.892 | 1767.686 |

**Supplementary Table 3.** Pairwise species *F*_ST_ for microsatellite and cpDNA markers.

|  | *C. gadorensis* | *C. pulvinata* |
| --- | --- | --- |
| *Microsatellites* |  |  |
| *C. pulvinata* | 0.074* |  |
| *C. sagredoi* | 0.096* | 0.054* |
| *cpDNA* |  |  |
| *C. pulvinata* | 0.322* |  |
| *C. sagredoi* | 0.588* | 0.520* |

Significant level: **P* < 0.05.

**Supplementary Table 4.** Pairwise population *F*_ST_ for microsatellites.

|  | LAR | MON | CAS | PEC | REV | RAG | SER | CAL | GAR |
| --- | --- | --- | --- | --- | --- | --- | --- | --- | --- |
| MON | 0.15 |  |  |  |  |  |  |  |  |
| CAS | 0.09 | 0.06 |  |  |  |  |  |  |  |
| PEC | 0.11 | 0.14 | 0.06 |  |  |  |  |  |  |
| REV | 0.11 | 0.13 | 0.08 | 0.08 |  |  |  |  |  |
| RAG | 0.14 | 0.15 | 0.12 | 0.08 | 0.15 |  |  |  |  |
| SER | 0.16 | 0.20 | 0.12 | 0.07 | 0.17 | 0.13 |  |  |  |
| CAL | 0.13 | 0.25 | 0.17 | 0.13 | 0.21 | 0.11 | 0.12 |  |  |
| GAR | 0.13 | 0.17 | 0.14 | 0.09 | 0.18 | 0.06 | 0.10 | 0.09 |  |
| SUF | 0.16 | 0.22 | 0.16 | 0.14 | 0.17 | 0.15 | 0.15 | 0.15 | 0.09 |

All values were significant at the 5% nominal level after sequential Bonferroni correction.

**Supplementary Table 5.** Mean recent migration rates (*m*) among the studied populations, estimated from six microsatellite loci using the BAYESASS program. Values on the diagonal (underlined) indicate the proportion of individuals in each generation that are not migrants. Simulations in BAYESASS show that in instances where there is no information in the data, the mean *m* and 95% confidence interval for data sets of 10 populations are 0.019 and 0.000–0.121, respectively.

|  | From |  |  |  |  |  |  |  |  | | |  | | |  |  |
| --- | --- | --- | --- | --- | --- | --- | --- | --- | --- | --- | --- | --- | --- | --- | --- | --- |
| To | LAR | MON | CAS | PEC | REV | RAG | SER | CAL | | | GAR | | | SUF | | |
| LAR | 0.983  (0.937–0.999) | 0.003  (0.000–0.024) | 0.002  (0.000–0.014) | 0.002  (0.000–0.016) | 0.002  (0.000–0.021) | 0.002  (0.000–0.014) | 0.002  (0.000–0.013) | 0.002  (0.000–0.013) | | 0.002  (0.000–0.015) | | | 0.002  (0.000–0.015) | | |  |
| MON | 0.002  (0.000–0.016) | 0.986  (0.947–1.000) | 0.002  (0.000–0.014) | 0.002  (0.000–0.014) | 0.002  (0.000–0.014) | 0.002  (0.000–0.014) | 0.001  (0.000–0.013) | 0.002  (0.000–0.013) | | 0.001  (0.000–0.012) | | | 0.002  (0.000–0.015) | | |  |
| CAS | 0.016  (0.000–0.078) | 0.019  (0.000–0.101) | 0.772  (0.669–0.993) | 0.051  (0.000–0.180) | 0.119  (0.000–0.306) | 0.004  (0.000–0.023) | 0.006  (0.000–0.034) | 0.004  (0.000–0.025) | | 0.004  (0.000–0.023) | | | 0.004  (0.000–0.024) | | |  |
| PEC | 0.002  (0.000–0.017) | 0.002  (0.000–0.018) | 0.002  (0.000–0.016) | 0.982  (0.934–1.000) | 0.002  (0.000–0.020) | 0.002  (0.000–0.014) | 0.002  (0.000–0.018) | 0.002  (0.000–0.014) | | 0.002  (0.000–0.013) | | | 0.002  (0.000–0.012) | | |  |
| REV | 0.002  (0.000–0.016) | 0.002  (0.000–0.013) | 0.002  (0.000–0.021) | 0.002  (0.000–0.014) | 0.985  (0.946–1.000) | 0.001  (0.000–0.012) | 0.001  (0.000–0.011) | 0.001  (0.000–0.012) | | 0.001  (0.000–0.012) | | | 0.002  (0.000–0.015) | | |  |
| RAG | 0.001  (0.000–0.011) | 0.002  (0.000–0.013) | 0.002  (0.000–0.014) | 0.002  (0.000–0.013) | 0.002  (0.000–0.016) | 0.986  (0.948–1.000) | 0.001  (0.000–0.014) | 0.002  (0.000–0.014) | | 0.002  (0.000–0.015) | | | 0.002  (0.000–0.012) | | |  |
| SER | 0.002  (0.000–0.020) | 0.002  (0.000–0.016) | 0.002  (0.000–0.019) | 0.003  (0.000–0.024) | 0.002  (0.000–0.016) | 0.003  (0.000–0.024) | 0.965  (0.874–0.999) | 0.014  (0.000–0.081) | | 0.004  (0.000–0.029) | | | 0.003  (0.000–0.021) | | |  |
| CAL | 0.003  (0.000–0.025) | 0.003  (0.000–0.023) | 0.003  (0.000–0.017) | 0.011  (0.000–0.052) | 0.003  (0.000–0.019) | 0.004  (0.000–0.033) | 0.005  (0.000–0.034) | 0.959  (0.893–0.996) | | 0.005  (0.000–0.032) | | | 0.003  (0.000–0.021) | | |  |
| GAR | 0.002  (0.000–0.016) | 0.002  (0.000–0.012) | 0.002  (0.000–0.015) | 0.002  (0.000–0.015) | 0.001  (0.000–0.011) | 0.003  (0.000–0.023) | 0.002  (0.000–0.015) | 0.003  (0.000–0.025) | | 0.981  (0.932–0.999) | | | 0.002  (0.000–0.017) | | |  |
| SUF | 0.002  (0.000–0.014) | 0.002  (0.000–0.015) | 0.001  (0.000–0.012) | 0.002  (0.000–0.016) | 0.002  (0.000–0.013) | 0.002  (0.000–0.017) | 0.002  (0.000–0.018) | 0.002  (0.000–0.017) | | 0.002  (0.000–0.016) | | | 0.982  (0.945–0.999) | | |  |

**Supplementary Table 6.** Median historical gene flow (*Nm*) among the studied populations, estimated from seven microsatellite loci using the formula 4*Nm* = *ΘM* (with *M* values obtained with MIGRATE-N). As suggested by Beerli (2006), the median is used instead of the mean since the latter is heavily influenced by outliers. In parentheses, 95% confidence interval. *Θ* is the mutation-scaled effective population size, as obtained from MIGRATE-N. Total immigration and emigration rates for each population were obtained by summing values of *Nm*.

| From |  |  |  |  |  |  |  |  |  |  |  |  |
| --- | --- | --- | --- | --- | --- | --- | --- | --- | --- | --- | --- | --- |
| To | Θ | LAR | MON | CAS | PEC | REV | RAG | SER | CAL | GAR | SUF | Total *Nm* (as immigration rates) |
| LAR | 3.500  (0-9.000) | --- | 1.488  (0-3.675) | 1.838  (0-4.200) | 1.313  (0-2.800 | 1.488  (0-4.025) | 1.663  (0-3.500) | 1.488  (0-3.500) | 1.663  (0-4.025) | 1.313  (0-2.975) | 1.488  (0-3.325) | 13.738 |
| MON | 3.500  (0-9.000) | 1.488  (0-3.500) | --- | 1.663  (0-4.200) | 1.488  (0-3.675) | 1.663  (0-4.025) | 2.013  (0-4.375) | 1.488  (0-3.500) | 1.488  (0-3.500) | 1.313  (0-3.150) | 1.663  (0-3.675) | 14.263 |
| CAS | 3.500  (0-9.000) | 1.838  (0-3.850) | 1.488  (0-3.325) | --- | 1.663  (0-3.675) | 1.663  (0-5.250) | 1.488  (0-3.325) | 1.488  (0-3.500) | 1.313  (0-2.975) | 1.488  (0-2.975) | 1.488  (0-3.500) | 13.913 |
| PEC | 3.500  (0-9.000) | 1.313  (0-2.625) | 1.488  (0-3.15) | 1.313  (0-2.800) | --- | 1.663  (0-3.675) | 1.488  (0-3.325) | 1.488  (0-3.150) | 1.663  (0-3.500) | 1.313  (0-2.800) | 1.313  (0-2.625) | 13.038 |
| REV | 3.500  (0-8.000) | 1.663  (0-3.500) | 1.488  (0-3.675) | 1.663  (0-4.200) | 1.663  (0-3.675) | --- | 1.313  (0-2.975) | 1.313  (0-2.800) | 1.488  (0-3.675) | 1.488  (0-2.975) | 1.488  (0-3.325) | 13.563 |
| RAG | 4.500  (0-10.000) | 1.463  (0-2.925) | 1.463  (0-3.150) | 1.463  (0-2.925) | 1.463  (0-3.150) | 1.463  (0-2.925) | --- | 1.463  (0-2.925) | 1.688  (0-3.150) | 1.688  (0-3.375) | 1.463  (0-2.925) | 13.613 |
| SER | 3.500  (0-9.000) | 1.488  (0-3.325) | 1.488  (0-3.150) | 1.313  (0-2.800) | 1.488  (0-3.150) | 1.313  (0-2.625) | 1.488  (0-3.150) | --- | 1.488  (0-3.500) | 1.663  (0-3.675) | 1.313  (0-2.975) | 13.038 |
| CAL | 3.500  (0-8.000) | 1.663  (0-3.675) | 1.138  (0-2.450) | 1.313  (0-2.625) | 1.488  (0-3.325) | 1.313  (0-2.800) | 1.663  (0-3.675) | 1.663  (0-3.325) | --- | 1.313  (0-2.975) | 1.313  (0-2.975) | 12.863 |
| GAR | 3.500  (0-9.000) | 1.313  (0-2.625) | 1.138  (0-2.275) | 1.138  (0-2.275) | 1.138  (0-2.450) | 1.138  (0-2.450) | 1.313  (0-2.800) | 1.138  (0-2.450) | 1.313  (0-2.800) | --- | 1.138  (0-2.450) | 10.763 |
| SUF | 4.500  (0-9.000) | 1.463  (0-2.925) | 1.463  (0-2.700) | 1.463  (0-2.925) | 1.463  (0-2.925) | 1.463  (0-2.925) | 1.463  (0-2.925) | 1.463  (0-2.700) | 1.463  (0-3.150) | 1.463  (0-2.925) | --- | 13.163 |
| Total *Nm* (as emigration rate) |  | 13.688 | 12.638 | 13.163 | 13.163 | 13.1630 | 13.888 | 12.988 | 13.563 | 13.038 | 12.663 |  |

Citation: Beerli, P. (2006). Comparison of Bayesian and maximum-likelihood inference of population genetic parameters. [*Bioinformatics*](https://www.ncbi.nlm.nih.gov/pubmed/16317072) 22, 341–345.
